# Precision and trueness verification study of an Atellica system

**DOI:** 10.1515/almed-2020-0096

**Published:** 2020-10-29

**Authors:** Alberto Vílchez Rodríguez, Julia González Cantó, Sara Esteve Poblador, Carmen Valldecabres Ortiz, Pedro L. Estela Burriel

**Affiliations:** Area of Biological Diagnosis, La Ribera University Hospital, Alzira, Spain

**Keywords:** biochemistry, immunochemistry, method comparison, method evaluation

## Abstract

**Objectives:**

Clinical laboratories should use only validated procedures. Precision is an important factor in the validation and verification of a new measurement procedure. Our objective was to verify the precision and trueness of different analysers used for the biochemical and immunochemical characterization of analytes.

**Methods:**

Advia 1800^®^, Immulite^®^2000 and CentaurXP^®^ analysers and the Atellica^®^Solution system were used. Five analytes were characterized biochemically, whereas another five analytes were characterized immunochemically. Imprecision was assessed using BioRad^®^ and Siemens^®^ control materials. Within-run and between-run imprecision were calculated by analysing three replicates of each control in a single run every day for five days. Bias was assessed using 40 samples of serum by the analysis of differences and linear regression.

**Results:**

The within-run and between-run imprecision values obtained with the new measurement procedure were lower than the ones claimed by the manufacturer for all the analytes studied. In the bias study, a proportional but not constant systematic error was observed in some analytes.

**Conclusions:**

The coefficients of variation obtained with Atellica^®^Solution verified both, the imprecision specifications claimed by the manufacturer and by the laboratory. The conditions of calibration should be revised for some parameters and a wider range of samples should be used.

## Introduction

Precision is essential for the correct interpretation and comparison of laboratory results.

The ISO 15189 standard for the certification of clinical laboratories establishes that laboratories should use only procedures that have been validated for their intended use. Thus, precision emerges as one of the most important metrological characteristics to be considered for the selection, implantation and validation of a measurement procedure [1].

The primary objective of this study was to verify the precision specifications claimed by the manufacturer in repeatability and intermediate conditions by calculating within-run and between-run imprecision. Secondary objectives included verifying the trueness (systematic error [SE]) of a novel measurement procedure by comparing it with the standard measurement procedure. Another objective was to verify if these measurement procedures are interchangeable and the reference values assigned to each analyte can be maintained.

## Materials and methods

The study was conducted in the Area of Biological Diagnosis of Hospital Universitario La Ribera, Alzira, Spain.

The clinical chemistry analysers employed were Siemens Healthineers: Advia 1800^®^ for clinical biochemistry, and Immulite^®^2000 and ADVIA CentaurXP^®^ for immunochemistry. The system evaluated was Atellica^®^Solution (biochemistry and immunochemistry), assuming the value yielded by the standard measurement procedures as the true value.

The analytes studied in Advia 1800^®^ were: Srm-calcium; c.sust., Srm-L-lactate dehydrogenase; c.cat. (LDH), Srm-ferritin; c.sust., Uri-albumin; c.mass. and total Uri-protein; c.mass.; in ADVIA CentaurXP^®^: Srm-25-OH-cholecalciferol; c.sust. (Vitamin D) and Uri-cortisol; c.sust.; and in Immulite^®^2000: Srm-N-terminal proBNP; c.sust. (NT-proBNP), Srm-pregnancy-associated plasma protein-A; c.arb. (PAPP-A) and Srm-Choriogonadotropin subunit beta; c.arb (β-HCG).

Both, the standard and the new biochemical characterization procedure are based on spectrophotometry. Changes were only observed in LDH reaction (for the standard pyruvate-to-l-lactate method and the new l-lactate-to-pyruvate method). Immunochemical characterization was performed by chemiluminescence in both, the standard and the new measurement method. Immunochemical characterization of ferritin was performed by immunoturbidimetry in the standard procedure and by chemiluminescence in the new procedure.

Commercially available BioRad^®^ control materials were used: Multiqual (calcium, ferritin and LDH; Liquichek Cardiac Markers (NT-proBNP); Immunoassay plus control (Vitamin D) and Urine Chemistry (total protein, albumin, and cortisol in urine). Siemens^®^ control materials included: IMMULITE^®^Systems PAA (PAPP-A) and IMMULITE^®^Systems FBC (β-HCG). Two levels of control were used for each analyte: a value near the cut-off point and a pathologic value. These values were similar to those used by the manufacturer in their imprecision study.

Imprecision was estimated according to SEQC guidelines, which are based on Clinical and Laboratory Standards Institute (CLSI) guidelines [2], [3]:

– Three replicates of each control were analysed in a single run every day for five days.
– Within-run imprecision (*CV*_*r*_):

$$s_{r}=\sqrt{\frac{\sum_{d=1}^{D}\sum_{i=1}^{n}{\left(X_{di}-X_{d}\right)}^{2}}{D\left(n-1\right)}}$$

$$CV_{r}=100.\frac{S_{r}}{X_{t}}$$

where *S*_*r*_=within-run deviation, *X*_*di*_=result for the replicate *i* on day *d*, *X*_*d*_=mean value of the day *d*, *D*=number of days (five), *n*=number of replicates per day (three) and *X*_*t*_=mean of all results.

– Between-run imprecision was calculated (*CV*_*T*_):

$$s_{b}^{2}=\frac{\sum_{i=1}^{D}{\left(X_{d}-X_{t}\right)}^{2}}{D-1}$$

$$S_{T}=\sqrt{\frac{n-1}{n}S_{r}^{2}+S_{b}^{2}}$$

$$CV_{T}=100.\frac{S_{T}}{X_{t}}$$

where *S*_*b*_=standard between-day deviation, *X*_*d*_=mean value of the day *d*, *X*_*t*_=total mean value, *D*=number of days (five), *S*_*T*_=between-run deviation, *S*_*r*_=within-run deviation and *n*=number of replicates per day (three).

– Within-run and between-run imprecision values were compared with the manufacturer’s specifications for the same range of concentrations. The estimated values had to be equal or lower than those reported by the manufacturer.

For the bias study, a total of 40 samples from different patients were analysed in five analytical runs using the two measurement procedures. After outliers were eliminated, cortisol and β-HCG were measured in a total of 38 samples. About 50% of the samples processed for each analyte showed values outside the range of reference. Concentrations were normally distributed along the measurement interval in all analytes.

The results of the bias study were analysed using two methods that are provided supplementary data [4]:

Analysis of differences: an analysis was performed using the differences between the result obtained with the new procedure (x) and the result obtained with the standard procedure (y). The mean value of each pair of results and relative percentage differences were calculated (*DR*). The difference between the results of the two procedures was expressed as a mean absolute (*Dm*) or relative (*DRm*) difference. Confidence intervals were calculated at 95%.
– Linear regression: “y” and “x” values were displayed, slope values were obtained (b) and the *y-*intercept was obtained (a) with the respective 95% confidence intervals.

## Results

Table 1 summarizes the values obtained in the within-run (*CV*_*r*_) and between-run (*CV*_*T*_) imprecision study, along with manufacturer’s values.

**Table 1:** Imprecision results (%).

			Imprecision, %			
Analyte	Control	Mean *x̄*	Manufacturer’s *CV_r_*	Estimated *CV_r_*	Manufacturer’s *CV_T_*	Estimated *CV_T_*
Srm-calcium; c.sust.	Level 1	1.54 mmol/L	1.2	0.7	1.7	0.7
	Level 3	3.27 mmol/L	0.6	0.5	0.7	0.7
Srm-L-lactate dehydrogenase; c.cat.	Level 1	189 U/L	0.9	0.7	1.0	0.9
	Level 3	403 U/L	0.7	0.4	1.0	0.6
Srm-ferritin; c.sust.	Level 1	92.1 pmol/L	1.4	1.4	4.2	1.4
	Level 3	143.8 pmol/L	1.3	1.1	4.4	1.0
Srm- N-terminal brain natriuretic pro-peptide; c.sust.	Level 1	15.4 pmol/L	2.3	1.8	3.9	3.7
	Level 3	377.8 pmol/L	1.8	1.7	3.7	2.8
Uri-albumin; c.mass.	Level 1	292 mg/L	1.4	1.0	3.6	0.9
	Level 3	409 mg/L	1.2	0.6	1.4	0.7
Uri-total protein; c.mass.	Level 1	121 mg/L	4.9	1.6	5.3	1.8
	Level 3	549 mg/L	1.2	0.7	1.9	0.6
Srm-25-OH-cholecalciferol; c.sust.	Level 1	60.3 nmol/L	5.0	4.8	8.0	4.3
	Level 3	226.9 nmol/L	1.8	1.6	3.2	2.5
Uri-cortisol; c.sust.	Level 1	19.5 nmol/day	2.6	2.3	8.9	4.1
	Level 3	63.7 nmol/day	5.5	4.7	9.6	7.9
Srm- pregnancy-associated plasma protein-A; c.arb.	Level 1	1.54 UI/L	2.7	2.2	3.4	2.3
	Level 3	4.17 UI/L	2.9	2.2	4.1	3.6
Srm-Choriogonadotropin subunit beta; c.arb.	Level 1	20.03 UI/L	1.5	1.1	2.5	2.4
	Level 3	177.7 UI/L	1.3	1.0	2.3	2.0

*CV*_*r*_, within-run coefficient of variation; *CV*_*t*_, between-run coefficient of variation.

Table 2 shows the data obtained in the bias study, which included an analysis of differences and a linear regression analysis.

**Table 2:** Results of the bias study.

		Serum							Urine		
		Srm-calcium	Srm-L-lactate dehydrogenase	Srm-ferritin	Srm- N-terminal brain natriuretic pro-peptide	Srm-pregnancy-associated plasma protein-A	Srm-Choriogonadotropin subunit beta	Srm-25-OH-cholecalciferol	Uri-cortisol	Uri-albumin	Uri-total protein
Analysis of differences	95% CI *Dm*	(−0.03; 0.13)	(200.17; 253.34)	(−27.29; −4.10)	(−74.73; 599.06)	(−1.68; −0.98)	(6.23; 9.20)	(−0.77; 1.34)	(−0.3; 6.14)	(−34.84; 130.39)	(−2.91; 6.88)
	95% CI *DRm*	(−0.25; 1.35)	(73.19; 78.69)	(−1.15; 3.22)	(4.68; 16.11)	(−30.51; −24.81)	(16.29; 21.69)	(−2.7; 7.62)	(−6.18; 20.48)	(−2.59; 16.97)	(−3.82; 19.08)
Linear regression	95% CI *a*	(−1.02; 0.87)	(-3.29; 77.06)	(−2.31; 17.85)	(−370.20; 116.17)	(−0.48; 0.33)	(−2.75; 3.62)	(0.61; 5.16)	(−10.14; −2.14)	(−30.25; 3.76)	(−4.24; 0.63)
	95% CI *b*	(0.91; 1.12)	(1.81; 2.22)	(0.92; 0.96)	(1.25; 1.43)	(0.71; 0.83)	(1.12; 1.29)	(0.78; 0.9)	(1.43; 1.91)	(1.13; 1.15)	(1.05; 1.07)
	*r*	0.898	0.902	0.997	0.955	0.945	0.956	0.899	0.848	0.999	0.999

95% CI, 95% interval of confidence; *Dm*, absolute differences; *DRm*, relative differences; *a*, *y*-intercept values; *b*, slope values; *r*, coefficient of correlation.

Based on the analysis of differences

– No constant or proportional systematic error (SE) was observed in total protein, albumin and urine cortisol; and calcium and vitamin D in serum, since the 95% CI of *Dm* and *DRm* included value 0.
– No constant SE (*Dm* 95% CI includes 0) but a proportional SE (95% CI *DRm* does not include 0) was observed in NT-proBNP in serum.
– A constant SE (*Dm* 95% CI does not include 0) but no proportional SE (*DRm* 95% CI includes 0) were observed for ferritin in serum.
– A constant SE (*Dm* 95% CI does not include 0) and a proportional SE (*DRm* 95% CI does not include 0) were observed in LDH, PAPP-A and β-HCG in serum.

Based on the linear regression analysis

– No constant or proportional SE was observed in the measurement of calcium in serum, as 95% CI of the *y*-intercept in the origin contains value 0 and the 95% CI of the slope contains value 1.
– No constant SE (95% CI *y-*intercept contains 0) but a proportional SE (95% CI slope does not contain 1) was observed in measurements of total protein and urine albumin; and PAPP-A, β-HCG, NT-proBNP, ferritin and LDH in serum.
– A constant SE (*y-*intercept 95% CI does not contain 0) and a proportional SE (95% CI slope does not contain 1) were observed in measurements of urine cortisol and vitamin D in serum.

**Figure 1:**
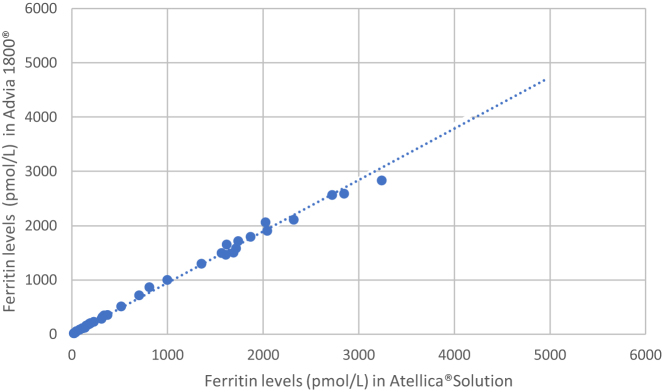
Linear regression analysis of ferritin in serum (pmol/L).

Figure 1 shows the linear regression study of ferritin in serum.

## Discussion

The precision study was performed under repeatability and intermediate conditions, with the latter being of more interest for the clinical laboratory. The coefficients of variation obtained with the Atellica^®^Solution system verify the imprecision specifications provided by the manufacturer for all measurement procedures and all the analytes studied. In addition, these results confirm compliance with the laboratory quality specifications (based on biological variability [5]) established by the laboratory for calcium, LDH, ferritin, albumin, total protein and NT-proBNP. No data of biological variability are currently available for vitamin D, PAPP-A, cortisol and β-HCG, although they complied with the requirements established in the state-of-the-art techniques, obtained with an external laboratory quality assessment program.

The bias study, based on the analysis of differences and linear regression analysis confirmed the absence of any significant constant or proportional SE for total protein, urine albumin, cortisol and ferritin, calcium and vitamin D in serum. However, no constant but a proportional SE was observed in NT-proBNP, LDH, β-HCG and PAPP-A in serum. For NT-proBNP, LDH and β-HCG, the slope was >1; therefore, the results obtained with the new measurement procedure can be proportionally higher than those obtained with the standard procedure. For PAPP-A, the slope was >1, and the results obtained with the new procedure can be lower than those obtained with the standard procedure.

The coefficients of correlation (r) obtained for linear regression analysis were <0.975 for NT-proBNP, LDH, PAPP-A, β-HCG, vitamin D and calcium in serum and urine cortisol; therefore, the interval of values should be extended using additional samples.

Different LDH results were obtained with the new measurement procedure. The inconsistencies detected may originate from the systematic error caused by methodological differences between the two procedures; therefore, different reference values should be employed.
